# A Unique Sequence Is Essential for Efficient Multidrug Efflux Function of the MtrD Protein of *Neisseria gonorrhoeae*

**DOI:** 10.1128/mBio.01675-21

**Published:** 2021-08-31

**Authors:** Mohsen Chitsaz, Vrinda Gupta, Benjamin Harris, Megan L. O’Mara, Melissa H. Brown

**Affiliations:** a College of Science and Engineering, Flinders University, Bedford Park, SA, Australia; b Research School of Chemistry, Australian National University, Canberra, ACT, Australia; Duke University School of Medicine

**Keywords:** RND protein, efflux pump, *Neisseria gonorrhoeae*, gonorrhea, multidrug resistance, MtrD protein, drug transport

## Abstract

Antimicrobial resistance in Neisseria gonorrhoeae has reached an alarming level, severely impacting the effective treatment of gonorrhea. Belonging to the resistance-nodulation-cell division (RND) superfamily of efflux transporters, the MtrD membrane protein of N. gonorrhoeae provides resistance to a broad range of antimicrobial compounds. A unique feature of MtrD is an 11-residue sequence (from N917 to P927 [N917-P927]) that connects transmembrane helices (TMS) 9 and 10; this sequence is not present in homologous RND proteins. This study explores the structural and functional roles of the N917-P927 region by means of mutant analysis and molecular dynamics simulations. We show that N917-P927 plays a key role in modulating substrate access to the binding cleft and influences the overall orientation of the protein within the inner membrane necessary for optimal functioning. Removal of N917-P927 significantly reduced MtrD-mediated resistance to a range of antimicrobials and mutations of three single amino acids impacted MtrD-mediated multidrug resistance. Furthermore, molecular dynamics simulations showed deletion of N917-P927 in MtrD may dysregulate access of the substrate to the binding cleft and closure of the substrate-binding pocket during the transport cycle. These findings indicate that N917-P927 is a key region for interacting with the inner membrane, conceivably influencing substrate capture from the membrane-periplasm interface and thus is essential for full multidrug resistance capacity of MtrD.

## INTRODUCTION

With an estimated 86 million new cases of gonorrheal infections in adults in 2016 caused by Neisseria gonorrhoeae, the longstanding disease gonorrhea remains a serious global public health problem (1), primarily due to ability of the bacterium to develop or acquire resistance mechanisms to clinically relevant antibiotics (2). Export of antibiotics across the cellular membrane via efflux pumps is one of the most efficient mechanisms used by N. gonorrhoeae, providing concomitant resistance to multiple antibiotics (3). Gonococci possess five drug efflux pump systems (4–6). Of these, the multiple transferrable resistance CDE (MtrCDE) system is the only transporter from the resistance-nodulation-cell division (RND) superfamily and is the most important efflux-mediated drug resistance mechanism (7, 8). Overexpression of MtrCDE imparts clinically significant levels of resistance to many important antibiotics used for treatment, such as β-lactams, and the currently used combined frontline treatment for gonorrhea of the third-generation cephalosporin ceftriaxone and macrolides such as azithromycin (4, 8–10). This can be mediated by *mtrR*, the transcriptional repressor of the *mtrCDE* operon (11, 12), through mutations in either *mtrR* or its promoter region (13–17) with the structural mechanism recently described (18). Furthermore, MtrCDE enables N. gonorrhoeae to persist in the presence of host-derived compounds, such as long-chain fatty acids, bile salts, hormones (e.g., progesterone) and cationic antimicrobial peptides that are found in human body sites frequently infected by N. gonorrhoeae, enhancing the ability of this pathogen to establish an infection or proliferate at mucosal surfaces (7, 19).

The MtrD component of the tripartite MtrCDE efflux system is a large protein of 1,067 amino acids, that, together with its closely related homologues (AcrB from Escherichia coli and MexB from Pseudomonas aeruginosa), belongs to the largely Gram-negative bacterial hydrophobe-amphiphile efflux-1 (HAE1) family of the RND superfamily (7, 20–22). The first crystal structure of MtrD revealed a homotrimer with symmetric monomers and an overall structure similar to those of homologous RND drug transporters (23). Each MtrD monomer has pseudosymmetric halves containing two domains: a transmembrane domain (TM) organized into 12 transmembrane α-helices (TMS) and a large periplasmic domain that can be subdivided into a porter domain and an MtrE docking domain (23). The porter domain is composed of four subdomains—PN1, PN2, PC1, and PC2—with three PN1 subdomains (one from each protomer) making up a central pore and stabilizing the trimeric organization. PC1 and PC2 create a cleft that opens to the periplasm likely allowing for periplasmic entry of substrates (23).

Recent cryo-electron microscopy (EM) structures of MtrD in complex with ampicillin and erythromycin revealed greater insight into the MtrD substrate translocation pathway and functional mechanism of drug efflux (24). In contrast to the first MtrD apo structure, these substrate-bound trimeric structures are asymmetric, similar to substrate-bound structures of AcrB and MexB (24–27). Each protomer adopts a distinct conformation resembling a three-step functionally rotating export cycle that is comprised of the access (A), binding (B), and extrusion (E) conformations (24–27). We recently described the functionally rotating transport mechanism of MtrD (28). As is the case for AcrB and MexB, the substrate permeation pathway through each MtrD protomer contains three possible entrance points, from the inner membrane (IM), from the periplasm, or via the central cavity (25–27, 29). Substrates then pass through the proximal and distal substrate recognition and binding sites and exit via the funnel-like channel within the MtrE docking domain (24–27, 29). The periplasmic entrance (also known as entrance channel 2 in AcrB) is located in the cleft formed between PC1 and PC2 and leads to the proximal pocket, likely collecting substrates found in the periplasm (25, 29, 30). Drugs that are partitioned in the membrane are expected to enter the pump via the IM entrance (also known as lateral entrance or entrance channel 1 in AcrB) that opens to the groove formed in the boundary between the TM and IM (25–27, 29). Surrounded by PN1, PN2, PC1, and PC2 β-sheets, the distal pocket is separated from the proximal pocket by a flexible switch loop or G-loop (29, 31, 32). These binding pockets are voluminous and formed by many hydrophobic, aromatic, polar, and charged residues (24–27, 29, 31). In the MtrD cryo-EM structures, ampicillin and erythromycin were both found in the distal pocket and only in the protomer adopting the binding conformation (24).

MtrD shares many, but not all, features with other bacterial HAE1 RND drug exporters (23, 27, 33). One of the unique features of MtrD is a sequence of 11 amino acids (N917 to P927 [N917-P927]) which is not present in homologous proteins. Using site-directed mutagenesis, molecular dynamics (MD) simulations, and resistance profiling, we explore the role of the N917-P927 region in MtrD-mediated multidrug resistance. We found that this unique region is essential for full multidrug efflux and resistance capacity of MtrD since its removal rendered N. gonorrhoeae cells susceptible to many antimicrobials, and three individual residues within it in particular are important for drug resistance. In addition, we show that N917-P927 may influence the accessibility of substrates to the proximal binding pocket and stabilization of the protein within the IM required for optimal function. Furthermore, deletion of N917-P927 in MtrD may dysregulate the access of substrate to the binding cleft and the propagation of conformational changes to the substrate binding pocket during the transport cycle.

## RESULTS

### Removal of N917-P927 significantly reduced MtrD-mediated resistance.

**FIG 1:**
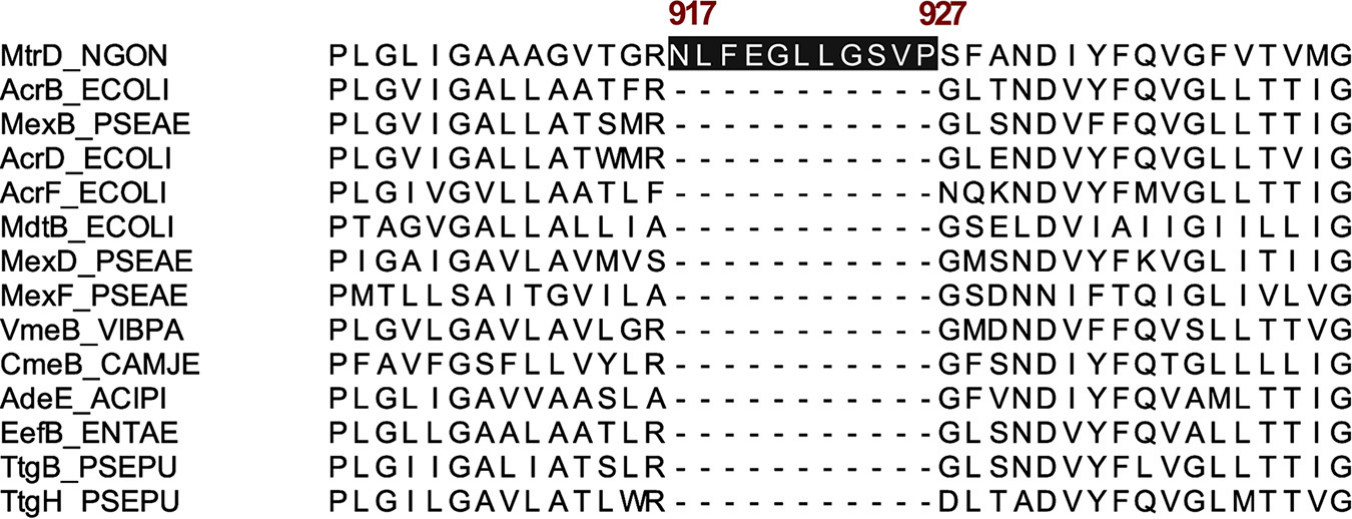
Protein sequences of selected members of RND proteins of the HAE1 family. Sequences were taken from the Transporter Classification Database (TCDB) (66, 67) and include MtrD of N. gonorrhoeae (Q51073), AcrB of E. coli (P31224), MexB of P. aeruginosa (P52002), AcrD of E. coli (P24177), AcrF of E. coli (P24181), MdtB of E. coli (P76398), MexD of P. aeruginosa (Q9HVI9), MexF of P. aeruginosa (Q9I0Y8), VmeB of Vibrio parahaemolyticus (Q2AAU3), CmeB of Campylobacter jejuni (AAL74245), AdeE of Acinetobacter sp. (Q8GKU1), EefB of Enterobacter aerogenes (Q8GC83), TtgB of Pseudomonas putida (O52248), and TtgH of P. putida (Q93PU4). Protein sequence alignment was performed using the NCBI protein blast tool and visualized using Ugene software. The N917-P927 sequence unique to MtrD is labeled in red.

**FIG 2:**
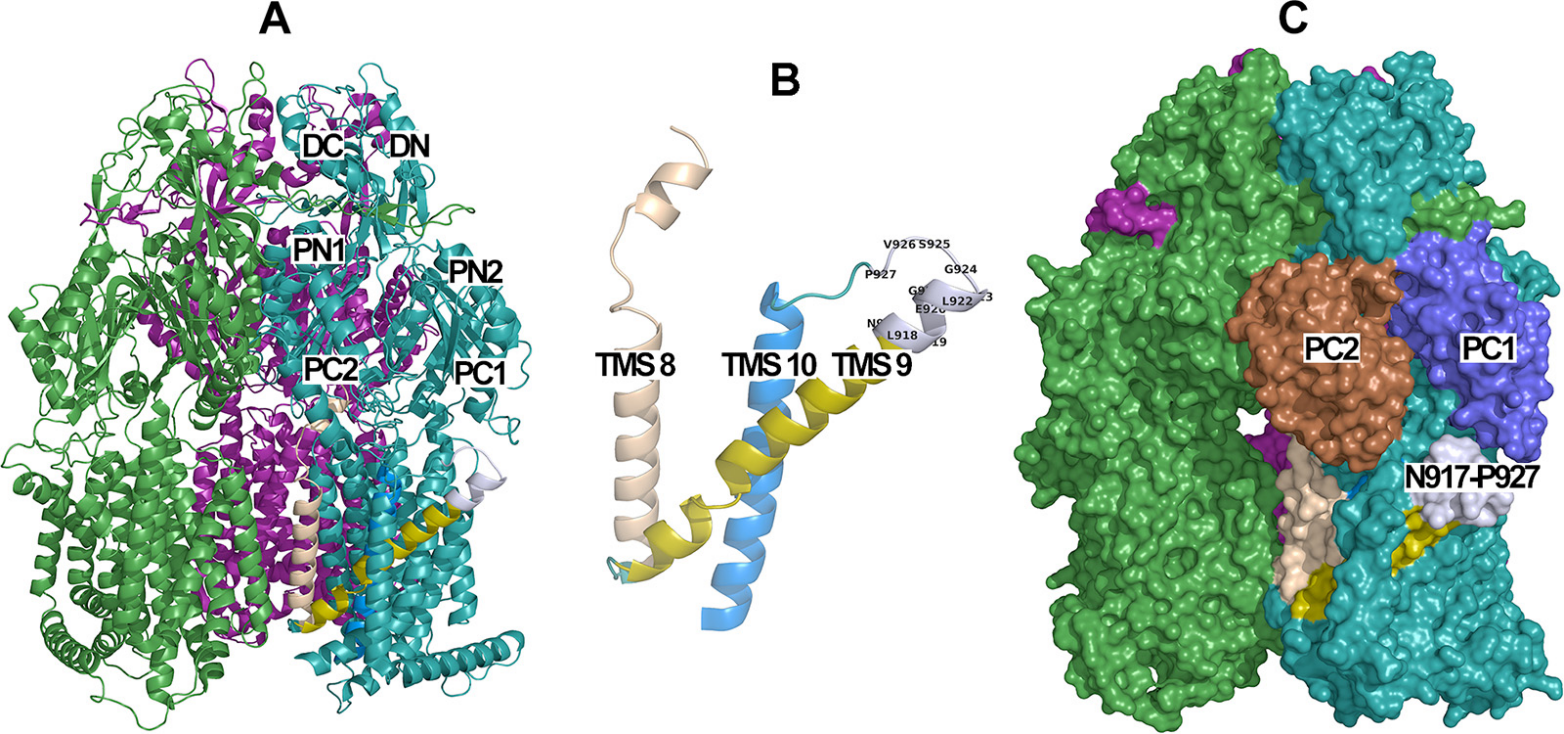
Secondary structure of the MtrD protein (PDB ID 6VKS). (A) Side view of the cryo-EM structure of the MtrD K823E variant. The three protomers are colored green (Access), teal (Binding), and magenta (Extrusion). The six subdomains of the porter domain are labeled in the B protomer. (B) Enhanced view of TMS 8 (beige), TMS 9 (gold), TMS 10 (marine blue), and N917-P927 (gray). (C) Surface representation of the K823E MtrD variant. Subdomains PC1 and PC2 in the binding protomer are shown in blue and russet, respectively. The position of the N917-P927 region at the bottom of the cleft opening between PC1 and PC2 is shown in gray.

Bioinformatics analyses revealed that the N917-P927 sequence is unique to the MtrD protein (Fig. 1). In addition, analyses of whole-genome sequences available in the NCBI database revealed that this 11-amino-acid sequence (NLFEGLLGSVP) is 100% conserved among 739 N. gonorrhoeae genomes, including the two multidrug-resistant clinical isolates, H041 and F89, that are highly resistant to the extended-spectrum cephalosporins ceftriaxone and cefixime (34–36). Furthermore, the sequence is also conserved in *mtrD* within other pathogenic and commensal *Neisseria* species (data not shown). This sequence forms a short α-helix extending from TMS 9 and continues to a loop connecting TMS 9 and TMS 10 (Fig. 2A and B) positioned directly beneath the binding cleft entrance formed by PC1 and PC2 (Fig. 2C). In addition, this region is linked via the N terminus of TMS 9 to the groove formed between TMS 8 and TMS 9, which has been proposed to facilitate collection of substrates from the outer leaflet of the IM through the IM entrance (entrance 1) in AcrB (Fig. 2A and B) (23, 37). The spatial arrangement of the N917-P927 region suggests that this local structure may play a role in facilitation of substrate uptake during a functional cycle. Furthermore, the observed high conservation of the N917-P927 sequence suggests that this region of MtrD does not play a substrate-specific interaction that would have evolved under specific antibiotic pressure.

As an initial step in determining the role of N917-P927, the sequence encoding this region was deleted from *mtrD* by PCR mutagenesis using primers MTRD_LF1 and MTRD_LR1 (see Table S1 in the supplemental material) and pGCC4-CL-*mtrD*_His6_ as the template DNA (38). The use of the pGCC4 vector allows for amplification of E. coli-based cloned *mtrD* or derivatives and subsequent integration of these determinants into the chromosome of a N. gonorrhoeae strain using a *Neisseria* insertion complementation system (NICS) (39). This provides the gonococcal cells with a recombinant *mtrD* allele expressed under *lac* regulation (38, 39). Thus, the resultant construct, pGCC4-CL-*mtrD*_His6_Δ(N917-P927), was recombined into the chromosome of N. gonorrhoeae strain KH15Δ*mtrD*Δ*norM*. This bacterial strain is a derivative of the N. gonorrhoeae KH15 strain known to bear a single bp deletion in the coinciding *mtrR* and *mtrC* promoter sequences, resulting in upregulation of the *mtrCDE* system and consequential increased levels of resistance to MtrD substrates (8). Since the NorM efflux pump shares some substrates in common with MtrD, the N. gonorrhoeae variant KH15Δ*mtrD*Δ*norM* with both the *norM* and *mtrD* efflux pumps deleted was constructed, producing a strain that was shown to be hypersusceptible to a broad range of MtrD-specific substrates (38, 40). Expression of recombinant wild-type (WT) *mtrD* from DNA integrated into this strain provided gonococcal cells with a fully functional MtrCDE efflux system mediating high levels of resistance to a broad range of antimicrobial compounds (38). Integration of the constructed CL-*mtrD*_His6_Δ(N917-P927) mutant produced the strain KH15Δ*mtrD*Δ*norM*-CL-*mtrD*_His6_Δ(N917-P927), which was confirmed by PCR using primers SCRNG1 and MTRDSF5 (see Table S1). SCRNG1 anneals to a location on the N. gonorrhoeae chromosome upstream of and outside the recombination site for the NICS of pGCC4 and MTRDSF5 anneals to the 3′ end of *mtrD* 167 bp upstream of its stop codon allowing verification of *mtrD* integration (38). Western blot analysis confirmed the presence of the MtrD Δ(N917-P927) deletion mutant protein in the membrane of KH15Δ*mtrD*Δ*norM*-CL-*mtrD*_His6_Δ(N917-P927) cells at a level comparable with WT (see Fig. S1). It has been shown previously that addition of a histidine tag on the C terminus of MtrD for monitoring expression levels and increasing the versatility of the system does not affect protein expression and resistance (38). Therefore, any change in resistance profile of this mutant compared to WT could be attributed to the constructed Δ(N917-P927) deletion. Consequently, the KH15Δ*mtrD*Δ*norM*-*mtrD*_His6_Δ(N917-P927) strain was tested for resistance against a panel of 10 compounds (listed in Table 1) using a standard MIC analysis method. These 10 compounds are known MtrD substrates and were selected because they give higher discrimination (≥4-fold difference in MIC) between the WT MtrD and the appropriate negative-control strain (KH15*ΔmtrD*Δ*norM* complemented with the NICS portion of the pGCC4 vector) (38). While the two macrolide antibiotics erythromycin and azithromycin are MtrD substrates with clinical importance, and our previous study has demonstrated excellent MIC differentiation between KH15Δ*mtrD*Δ*norM* and its parental strain KH15, the use of the erythromycin resistance cassette present in pGCC4 as a selection marker precluded testing constructed MtrD mutant derivatives against these antibiotics (38, 39). MIC analysis revealed that the deletion of N917-P927 did not totally abrogate but reduced resistance to all 10 tested compounds from a minimum of 1.3-fold for cholic acid to a maximum of >128-fold for nonoxynol-9 (Table 1). These data indicate that the N917-P927 sequence is necessary for complete and efficient activity of the MtrD protein.

**TABLE 1:** Antimicrobial resistance of N. gonorrhoeae strain KH15*ΔmtrDΔnorM* expressing MtrD derivatives

Mutation	MIC (μg/ml)^*a*^									
	Detergent		Antibiotic			CAMP	Dye		Bile acid	Hormone
	N-9	TX-100	RIF	NOV	OXA	PMB	ET	CV	CHO	PRO
WT^*b*^	≥4,096	≥4,096	0.125	1	4	100	0.25	2	400	80
Δ(N917-P927)	64	128	0.03	0.5	2	50	0.125	1	300	40
N917C	32	64	0.015	0.25	1	50	0.06	0.5	300	40
L918C	≥4,096	≥4,096	0.125	1	4	100	0.25	2	400	80
F919C	≥4,096	≥4,096	0.125	1	4	100	0.25	2	400	80
E920C	≥4,096	≥4,096	0.06	1	4	100	0.25	1	300	80
G921C	≥4,096	≥4,096	0.125	1	4	100	0.25	2	400	80
L922C	≥4,096	≥4,096	0.125	1	2	100	0.25	2	400	80
L923C	≥4,096	≥4,096	0.125	1	4	100	0.25	2	400	80
G924C	32	64	0.015	0.25	2	50	0.06	0.5	200	40
S925C	≥4,096	≥4,096	0.125	1	4	100	0.25	2	400	80
V926C	≥4,096	≥4,096	0.125	1	4	100	0.25	2	400	80
P927C	32	64	0.03	0.25	1	50	0.06	0.5	300	40
11Ala	ND	32	ND	ND	0.06	ND	ND	ND	ND	ND
N917K	16	32	0.015	0.06	0.25	50	0.06	0.125	100	40
N917Q	32	64	0.015	0.25	0.5	50	0.06	0.5	200	40
N917S	64	128	0.06	1	2	75	0.125	1	300	80
P927G	≥4,096	≥4,096	0.125	1	4	100	0.25	2	400	80
Negative control^*c*^	16	32	0.008	0.03	0.06	50	0.06	0.125	100	20

a All MIC data are representative of three or more independent experiments. Abbreviations: CAMP, cationic antimicrobial peptide; CHO, cholic acid; CV, crystal violet; ET, ethidium; N-9, nonoxynol-9; NOV, novobiocin; OXA, oxacillin; PMB, polymyxin B; PRO, progesterone; RIF, rifampin; TX-100, Triton X-100; ND, not determined.

b Recombinant cysteine-less MtrD variant with a 6-histidine affinity tag at C terminus (CL-MtrD_His6_).

c Strain KH15*ΔmtrD*Δ*norM* transformed with pGCC4 empty vector expressing no MtrD [KH15*ΔmtrD*Δ*norM* (NICS) strain].

10.1128/mBio.01675-21.1 TABLE S1 Oligonucleotides used in this study. Download Table S1, DOCX file, 0.06 MB. Copyright © 2021 Chitsaz et al. 2021 Chitsaz et al. https://creativecommons.org/licenses/by/4.0/ This content is distributed under the terms of the Creative Commons Attribution 4.0 International license.

10.1128/mBio.01675-21.2 FIG S1 Western blot image showing expression of constructed MtrD mutants. Equal amounts of protein from isolated membranes of N. gonorrhoeae KH15*ΔmtrD*Δ*norM* expressing various constructs were separated on a polyacrylamide gel and transferred to a polyvinylidene difluoride membrane. MtrD proteins (identified by a protein band at ∼100 kDa as the predicted molecular weight was 113.62 kDa) were immunologically detected using a rabbit anti-6His tag antibody that binds to the C-terminal His tag on MtrD. Note that the Western blot image is a combination of multiple individual experiments and includes (from left to right): negative control (−ve), MtrD KH15*ΔmtrD*Δ*norM* (NICS), that carries pGCC4 and lacks MtrD; cysteine-less MtrD C491A positive control; 917-927 MtrD deletion derivative; 11 individual cysteine replacement mutants, the 11 alanine replacement MtrD mutant [11 A (917-927)], and four individual amino acid replacement MtrD mutants. Download FIG S1, TIF file, 0.9 MB. Copyright © 2021 Chitsaz et al. 2021 Chitsaz et al. https://creativecommons.org/licenses/by/4.0/ This content is distributed under the terms of the Creative Commons Attribution 4.0 International license.

### Integrity of the N917-P927 sequence is required for efficient efflux activity of the MtrD protein.

To investigate whether the length or the specific residue composition of the N917-P927 sequence is an important factor for full function of the MtrD protein, the 11-amino-acid sequence was replaced with all alanines by site-directed mutagenesis using 11Ala F and 11Ala R primers (see Table S1) and pGCC4-CL-*mtrD*_His6_(ΔN917-P927) as the template DNA. The resultant 11 alanine replacement mutant 11A(917-927) was integrated into the chromosome of the KH15Δ*mtrD*Δ*norM* strain, and PCR using the primers SCRNG1 and MTRDSF5 confirmed the existence of this mutant in the chromosome. Using Western analysis, cells expressing the *mtrD* mutant, were found to produce protein at a very low level, and MIC assays against Triton X-100 and oxacillin showed no resistance, exhibiting a resistance profile similar to that of the negative control (Table 1; see also Fig. S1). Thus, this mutant was not experimentally analyzed any further. A second mutant was similarly designed that replaced the N917-P927 sequence with a stretch of 11 glycine residues 11G(917-927). However, construction of this mutant was unsuccessful possibly due to lethality. Together, these data indicated that the presence of the protein backbone alone (glycine substitution) or truncated side chains (alanine substitution) is not sufficient to support production of a fully active MtrD variant.

### Four residues in the N917-P927 region play an important role in MtrD activity.

As mentioned above, the positioning of the N917-P927 region suggests it has structural and functional roles, e.g., by facilitation of substrate uptake during a functional cycle. Therefore, it was proposed that some residues in this region may be involved in mechanisms that help substrate uptake from the IM and are important for MtrD activity. To identify the functional importance of individual residues within this region, each of the 11 residues was individually substituted with cysteine; cysteine was selected to facilitate later studies on the location/inhibition of substituted residues (41) and allows characterization of properties such as charge, polarity, or hydrophobicity linked to the target residue side chain. The 11 mutants were generated by site-directed mutagenesis using the primers listed in Table S1 and pGCC4-CL-*mtrD*_His6_ as the template DNA. All 11 MtrD mutants were expressed in KH15Δ*mtrD*Δ*norM* cells at levels comparable to that of the WT, as judged by Western blotting (see Fig. S1), indicating that the amino acid substitutions did not affect MtrD protein stability and membrane insertion. MIC analyses revealed that cells expressing six of the MtrD mutants (L918C, F919C, G921C, L922C, L923C, S925C, and V926C) retained resistance profiles similar to that of the WT with no change in MICs for any of the 10 tested compounds (Table 1). In contrast, three MtrD mutants (N917C, G924C, and P927C) showed a reduction in resistance to all compounds with the greatest decrease (>128-fold) to the nonionic and amphiphilic surfactant Triton X-100 and the spermicide nonoxynol-9 (Table 1), demonstrating that these three residues are critical for efficient MtrD-mediated resistance against a broad range of substrates. In addition, mutation of a fourth residue (E920C), affected resistance to rifampin, crystal violet, and cholic acid (Table 1). These data raised questions about the possible role(s) played by these residues in the MtrD efflux process. Specifically, do they directly bind MtrD substrates or alternatively interact with neighboring residues to indirectly affect transport function through another mechanism, e.g., by altering the volume of the nearby substrate entrance? To address these questions, we conducted additional site-directed mutagenesis and MD simulations.

To ascertain the role played by N917, and to determine whether there were side chain requirements/limitations in this position, we aimed to replace the polar noncharged side chain of asparagine with amino acids of both similar and different side chain properties. N917 was replaced with two polar noncharged residues (glutamine and serine) and the positively charged lysine. In addition, replacement of asparagine with a negatively charged residue (aspartic acid) and an amphipathic and bulky amino acid (tryptophan) was attempted; however, construction of these two mutants was unsuccessful. Western blot analyses confirmed the expression of N917K, N917Q, and N917S proteins in the membranes of KH15Δ*mtrD*Δ*norM* cells expressing these mutant MtrD proteins at levels comparable to that of WT (see Fig. S1). Assays determined that resistance was not fully maintained by replacing N917 with either glutamine or serine although the N917S derivative mediated higher levels of resistance compared to the original N917C mutation (Table 1). These data indicate that side chain properties of asparagine are required at position 917. The MtrD N917K derivative exhibited reduced resistance to many of the tested compounds even more than that by the N917C mutation, with a resistance capacity for nonoxynol-9, Triton X-100, ethidium, cholic acid, and polymyxin B only at levels comparable to the negative control (Table 1).

**FIG 3:**
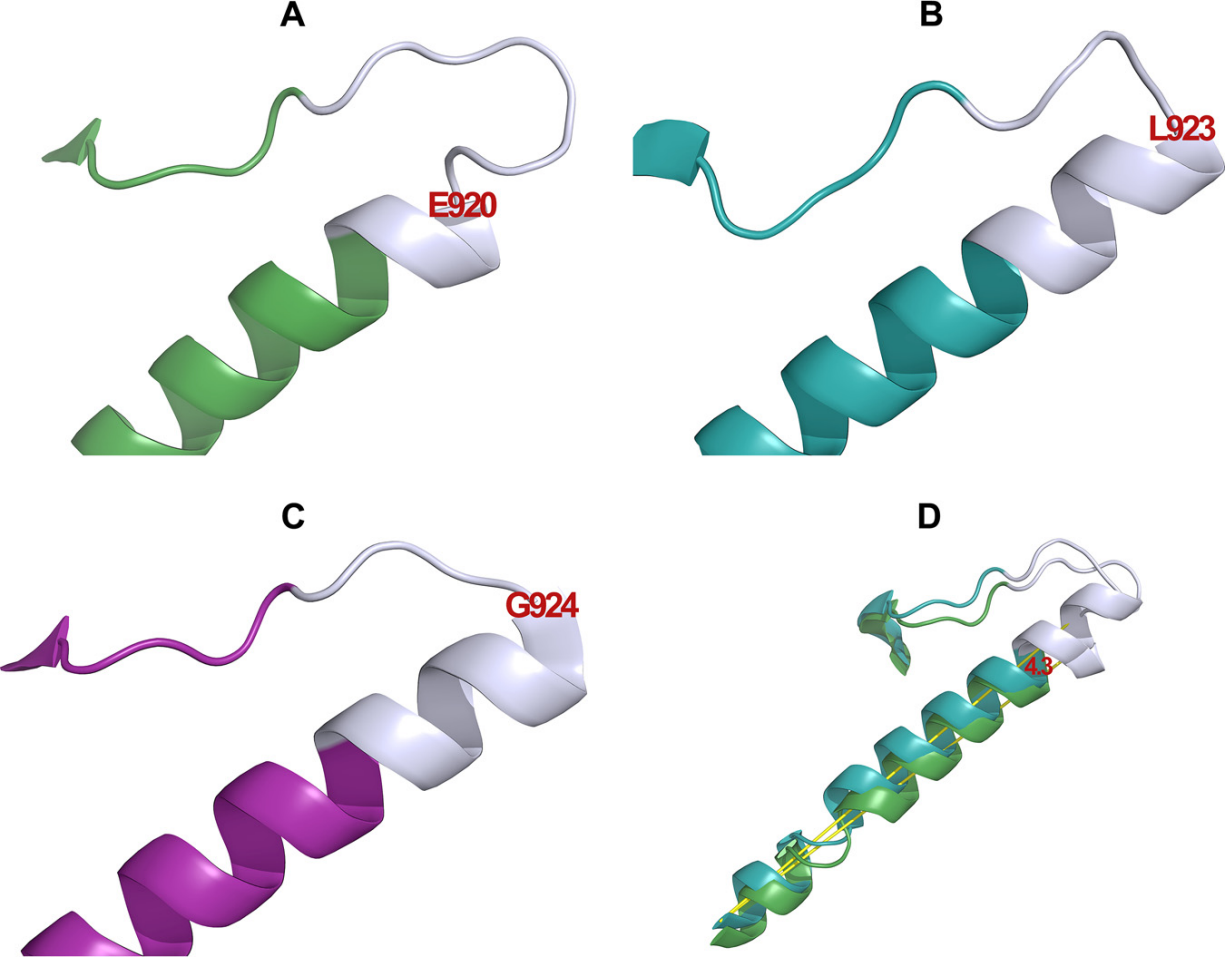
Secondary structure of the MtrD protein in complex with ampicillin (PDB ID 6VKS). The C terminus of TMS 9 and the N917-P927 is shown in cartoon representation. Each helix is colored according to its protomer: Access (green), Binding (teal), and Extrusion (magenta). N917-P927 is shown in gray in all pictures. (A) TMS 9 in the A protomer α-helix extends up to E920. (B) TMS 9 in the B protomer extends up to L923 forming two full turn α-helix turn in this region. (C) TMS 9 in the E protomer extends up to G924. (D) Superimposition of the A protomer and the B protomers (root mean square deviation of 1.6 Å). Residues 893 to 935 encompassing TMS 9 are shown in cartoon representation and the 4.3° angle formed between the C-alpha atoms of residues E920 and S893 is illustrated in yellow.

We hypothesized that both G924 and P927 are residues required for optimal/efficient efflux function of MtrD through providing structural flexibility (glycine) or constraining backbone geometry (proline) within the loop region. Both proline and glycine are associated with a propensity to destabilize α-helices, promoting the formation of unstructured loops and modifying substrate capture from the IM. Interestingly, in the MtrD extrusion protomer, G924 is located at a position that corresponds to a break in the α-helix (Fig. 3). To test these hypotheses, we designed mutagenesis strategies to create the MtrD mutants G924P and P927G. Construction of the G924P mutant was not successful likely due to lethality in E. coli cells; however, the MtrD P927G derivative was obtained and shown to be expressed at a level comparable to that of WT (see Fig. S1). MIC analysis of MtrD P927G in KH15Δ*mtrD*Δ*norM* cells indicated that the proline at this position could be replaced by glycine since it retained full resistance capacity (Table 1). We postulate that this may be due to the backbone conformational flexibility of glycine, which allows it to adopt backbone dihedral conformations that span the region of the Ramachandran plot occupied by proline (42). Together, these data showed that N917, G924, and P927 play important roles in MtrD-mediated multidrug resistance.

### Structural analysis and dynamics of the N917-P927 region.

The ampicillin-bound cryo-EM structure of MtrD (PDB ID 6VKS) revealed that the conformation of the N917-P927 region at its N terminus is different in the A, B, and E protomers. In the A protomer the α-helix of TMS 9 is shorter than in the B and E protomers (Fig. 3A to C). In the A protomer the TMS 9 α-helix extends to E920, while in the B protomer it extends to L923 and is further extended in the E protomer to G924 (Fig. 3A to C). These changes correspond to a change in the overall tilt angle of TMS 9 within the bilayer between these three conformations (Fig. 3D).

**FIG 4:**
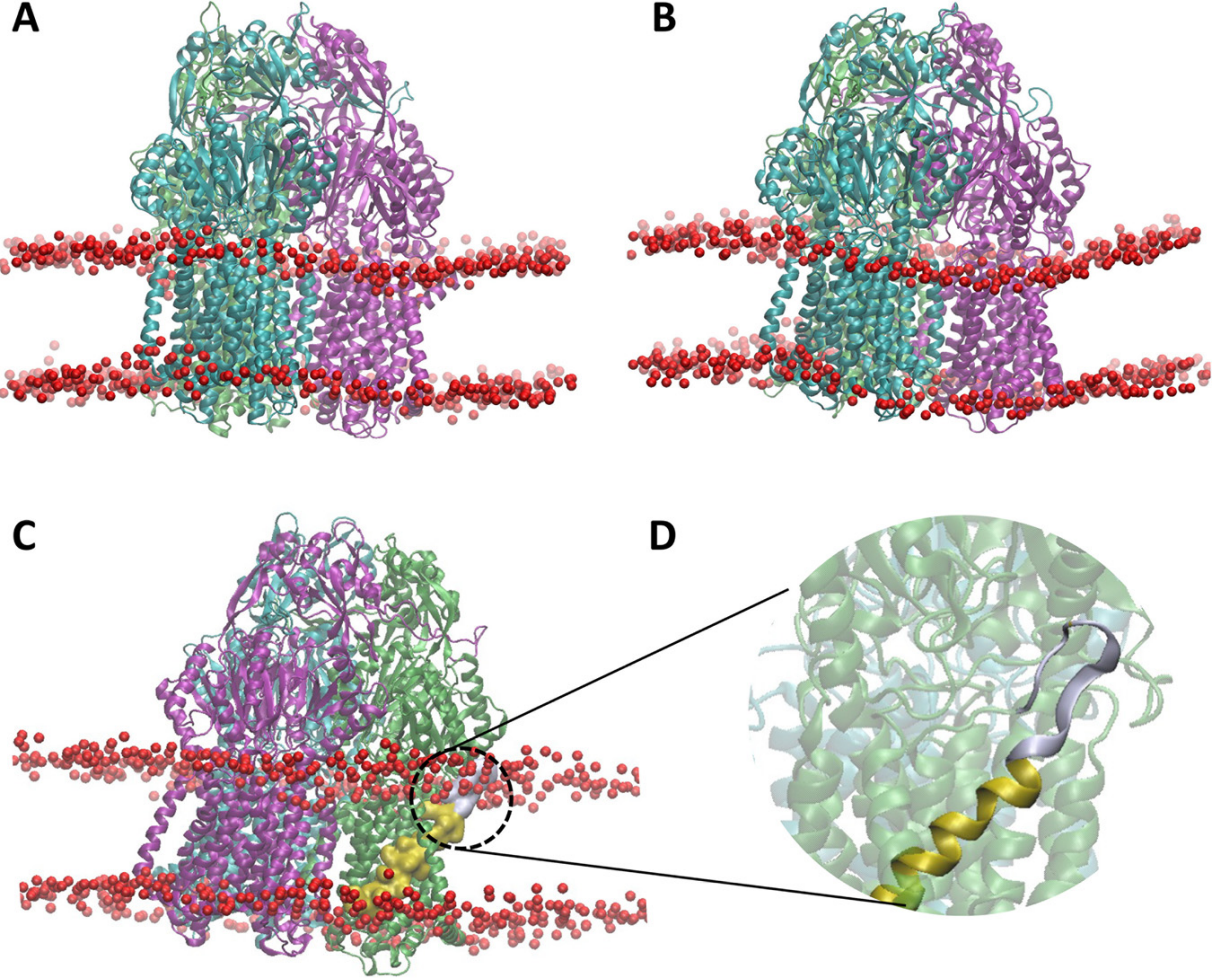
MD simulations showing the position of MtrD within the membrane. The three MtrD protomers are shown in ribbon representation and colored Access (green), Binding (teal), and Extrusion (magenta). The lipid phosphate head groups of the membrane are shown as red beads. (A to C) Positions of the WT trimer (A), Δ(N917-P927) trimer (B), and 11G(917-927) trimer (C) within the membrane. (D) Closeup view of the TMS 9 and N917-P927 regions in the Access protomer of the 11G(917-927) MtrD mutant. In panels C and D, TMS 9 is gold and N917-P927 is gray.

To better understand the impact of N917-P927 and the orientation of the TMS 9 helix on MtrD function, the ampicillin-bound cryo-EM structure of MtrD was embedded in a solvated neisserial membrane model (38) and MD simulations were carried out for 500 ns in triplicate (Fig. 4A). MD simulations were also performed on the Δ(N917-P927), 11G(917-927) variants, and an MtrD triple mutant (N917C/G924C/P927C), containing concurrent N917C, G924C, and P927C substitutions (Fig. 4B and C).

MD simulations of the WT MtrD trimer showed that N917-P927 of the A protomer lay within the membrane, positioned at the periplasmic membrane interface. In comparison, N917-P927 of the B and E protomers extended above the membrane into the periplasm (see Fig. S2A to C). The amino acid sequence of N917-P927 (NLFEGLLGSVP) is aliphatic, supporting its preferential location at the membrane-water interface.

10.1128/mBio.01675-21.3 FIG S2 Representative MD simulation snapshot of the position and orientation of the TMS 9 (yellow) and N917-P927 (grey) region in relation to the N. gonorrhoeae membrane surface. Position and orientation of TMS 9 and N917-P927 is shown for the Access (green) (A), Binding (teal) (B), and Extrusion (magenta) (C) protomers of the WT MtrD protein and the Access (green) (D), Binding (teal) (E), and Extrusion (magenta) (F) protomers of the Δ(N917-P927) MtrD mutant. Download FIG S2, TIF file, 2.1 MB. Copyright © 2021 Chitsaz et al. 2021 Chitsaz et al. https://creativecommons.org/licenses/by/4.0/ This content is distributed under the terms of the Creative Commons Attribution 4.0 International license.

In replicate 500-ns simulations of WT MtrD, the position and relative orientations of the TMS 9 extension, N917-P927, changed relative to the starting cryo-EM structure. In all three protomers of the cryo-EM structure, N917-P927 did not form close contacts with other regions of the protein. Within the first 50 ns of simulation, N917-P927 formed contacts with residues in the periplasmic domain, which differed between the A, B, and E protomers.

**FIG 5:**
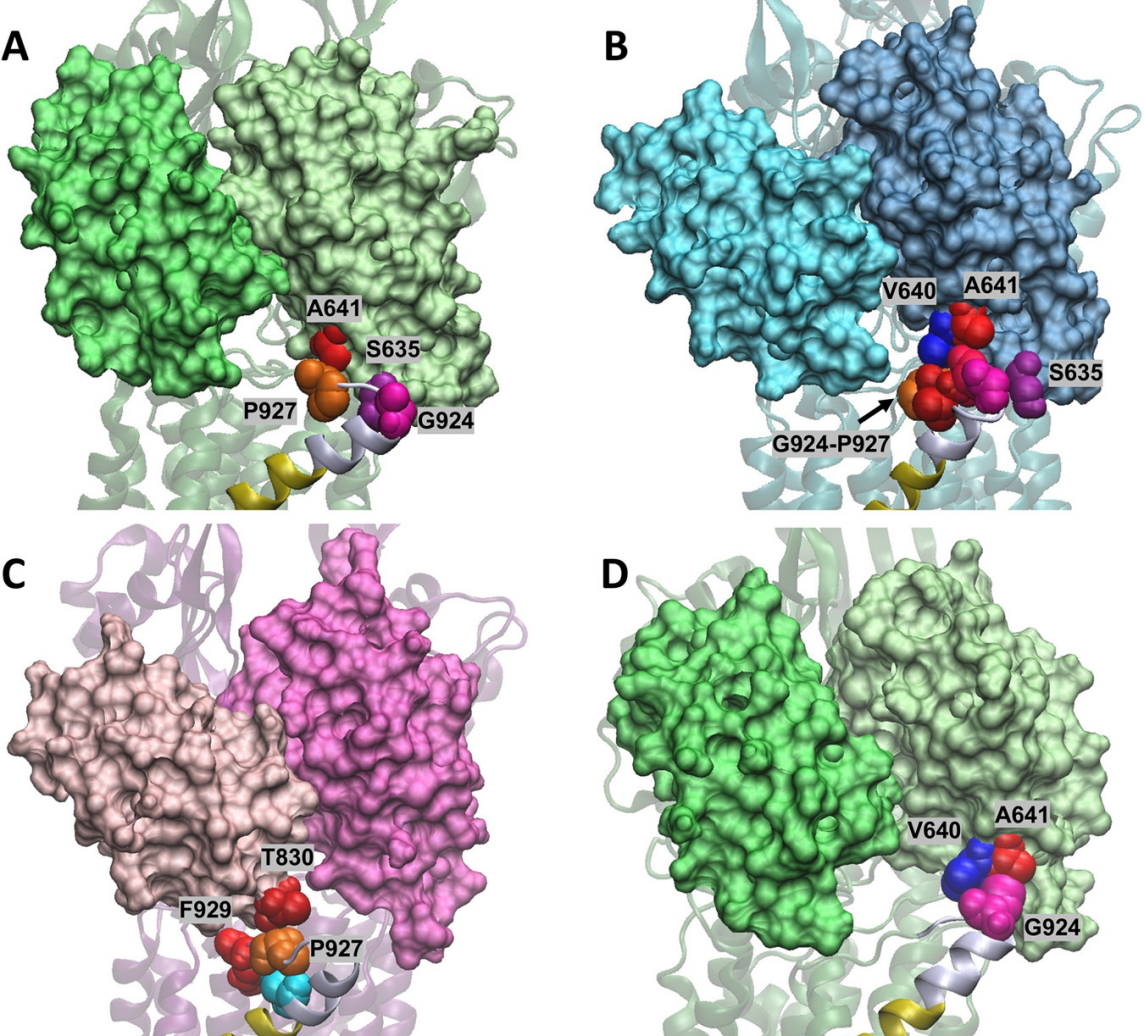
MD simulation analysis of interactions between the N917-P927 region and the MtrD periplasmic domain. TMS 9 is in gold and the N917-P927 region is in gray ribbon representation. Interactions are shown for the Access (green) (A), Binding (teal) (B), and Extrusion (magenta) (C) protomers in the WT MtrD protein and the Access (green) protomer (D) of the N917C/G924C/P927C triple mutant MtrD protein. The PC1 (right) and PC2 (left) domains are shown in surface representation. Interacting residues (labeled on the figure) are shown in Van der Waals (VDW) rendering and are colored by residue identity.

In the A protomer, N917-P927 remained close to the PC1 domain near the membrane interface. In particular, G924 was in close proximity to S635 from the PC1 domain, while P927 was positioned near to A641 (Fig. 5A). In the B protomer, N917-P927 interacted with the PC1 domain slightly higher in the binding pocket than in the A protomer and directly contacts it to form a protein-protein interface (Fig. 5B). In all three replicate simulations, the G924-P927 region formed protein-protein contacts in the vicinity of V640, A641, and S635. Compared to the A protomer, the change in position of N917-P927 effectively narrowed the binding cleft entrance and prevented the efficient entry of substrates (Fig. 5B). The precise nature of the contacts between G924-P927 and V640, A641 and S635 varied by replicate simulation. Notably, in one replicate simulation, the TMS 9 helical extension reoriented so that residues 917 to 927 adopted an elongated conformation in which G924 extended vertically into the base of the binding cleft to form a protein-protein contact with V640. In the remaining two replicate simulations, the conformation is less extreme. In all three replicate simulations, these interactions partially occluded the periplasmic binding cleft entrance. In the E protomer, N917 interacts with F929, while P927 interacts with T830 on the PC2 side of the binding cleft (Fig. 5C), protruding into the binding cleft to physically prevent substrate entry from both the periplasm and the IM substrate entrance.

MD simulations were then performed to assess the impact of the cysteine replacements at positions that were found to be important for MtrD function. Based on the underlying hypothesis that individual mutations within a protein are unlikely to perturb the overall secondary structure, a triple MtrD mutant, containing concurrent N917C, G924C, and P927C mutations (N917C/G924C/P927C) was developed. No steric clashes were observed in MtrD upon mutation of N917, G924, or P927 to cysteine. MD simulations showed that in contrast to the WT, for the triple mutant both the position and orientation of residues 917 to 927 in each protomer fluctuated and protruded above the membrane interface into the periplasm (see Fig. S3A to C). Interactions between the N917-P927 region and the rest of the protein are most altered in the A protomer of the triple cysteine mutant. In the A protomer, the N917-P927 loop extends to form a hairpin oriented into the binding cleft. Here, G924C interacts with V640 and A641 from the PC1 domain (Fig. 5D). This interaction extends further into the binding cleft than in simulations of WT MtrD, in which G924 interacts with S635 below the level of V640 and A641. The deeper penetration of G924C into the binding cleft entrance partially occluded the periplasmic entrance to the binding cleft. We hypothesize that this prevented the entry of some substrates through the proposed periplasmic entrance of the mutant MtrD A protomer. In the B protomer of the triple cysteine MtrD mutant, G924C interacts with S635 from the PC1 domain, anchoring N917-P927 at the lower right-hand entrance of the binding cleft. This interaction was also observed in the B protomer for the WT simulations but was more persistent throughout the simulations of the triple cysteine mutant. In the E protomer of the triple mutant, N917C formed contacts with F929, while P927C interacted with T830 on the PC2 side of the binding cleft, as was observed in the WT E protomer. We hypothesize that the P927C mutation decreases backbone dihedral angle constraints associated with the presence of proline, resulting in greater backbone mobility. The noticeable alteration in the positioning of the N917-P927 region in the A protomer of the triple cysteine mutant compared to the WT protein suggests the mutated residues N917, G924, and P927 play a key role in controlling substrate entry. The higher positioning of N917-P927 in the binding cleft of the triple cysteine mutant partially occluded the binding cleft entrance, preventing the entry of some substrates in the mutant MtrD A conformation. This restriction in substrate entry supports the decreased resistance profile experimentally observed upon mutation of these residues. Bioinformatics analysis of MtrD proteins in the NCBI database revealed that T830 and N929 are very highly conserved (100% amino acid identity), while S635 (63%), V640 (24%), and A641 (55%) are less conserved within N. gonorrhoeae strains. A similar homology trend is seen in MtrD present in strains of the closely related species Neisseria meningitidis. The conservation of these PC1/PC2 domain residues supports the MD simulations and the proposition that they inter-react with TMS 9 in MtrD efflux.

10.1128/mBio.01675-21.4 FIG S3 Representative snapshot of the position and orientation of the TMS 9 (yellow) and N917-P927 (grey) region from MD simulations in relation to the N. gonorrhoeae membrane surface. Position and orientation of TMS 9 and N917-P927 is shown for the Access (green) (A), Binding (teal) (B), and Extrusion (magenta) (C) protomers of the N917C/G924C/P927C triple mutant and the Access (green) (D), Binding (teal) (E), and Extrusion (magenta) (F) protomers of the 11G(917-927) mutant. Download FIG S3, TIF file, 2.1 MB. Copyright © 2021 Chitsaz et al. 2021 Chitsaz et al. https://creativecommons.org/licenses/by/4.0/ This content is distributed under the terms of the Creative Commons Attribution 4.0 International license.

Simulations of the Δ(N917-P927) MtrD mutant showed the terminal region of TMS 9 was located at the periplasmic membrane-water interface in the A and B protomers and protruded slightly above the membrane into the periplasm in the E protomer (see Fig. S2D to F). Simulations of the Δ(N917-P927) MtrD protein, showed TMS 9 and TMS 10 did not associate with the periplasmic entrance to the binding cleft and a greater localized curvature in the membrane around MtrD was observed (Fig. 4A and B), suggesting that N917-P927 plays a role in the stability and orientation of MtrD within the membrane. Destabilization of the protein-lipid interface of MtrD upon deletion of N917-P927 supports the hypothesis that this region plays a key role in stabilizing MtrD within the membrane.

Simulations of the 11G(917-927) MtrD mutant show the 11G(917-927) region protruded above the membrane into the periplasm in the A and B protomers (see Fig. S3D and E). In the E protomer, 917-927Gly lay at the periplasmic membrane interface (see Fig. S3F). Consistent with the mutation to glycine, there was a loss of α-helical secondary structure in 11G(917-927) (Fig. 4D) across all three protomers. There was also a noticeable tilting of the 11G(917-927) MtrD mutant within the membrane during the 500-ns replicate MD simulations (Fig. 4C), again indicating a destabilization in the protein-lipid MtrD interface, as was seen in the Δ(N917-P927) mutant. The overall changes in the localization and stability of N917-P927 between the MtrD mutants may underpin the limited activity of the transporter seen in the MIC analyses.

### Deletion of the N917-P927 region alters progesterone uptake.

**FIG 6:**
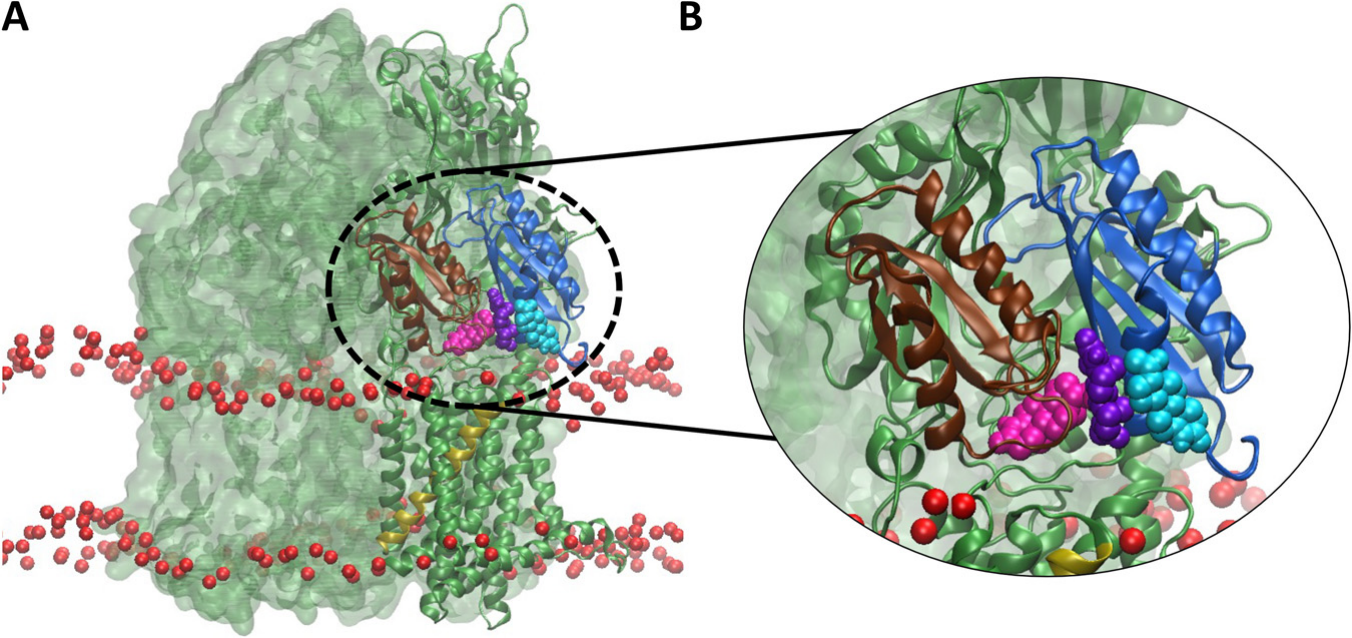
MD simulations of progesterone flooding of the Δ(N917-P927) mutant of MtrD (PDB ID 4MT1). Phosphate groups of the N. gonorrhoeae phospholipid inner membrane are shown as red beads. All three MtrD protomers are green, and TMS 9 is gold. The binding cleft of the PC1 domain is dark blue and the PC2 domain is brown. Three progesterone molecules (magenta, violet, and cyan) within the binding cleft entrance are shown in VDW representation. (A) Representative view showing multiple progesterone molecules in the MtrD binding cleft after 250 ns of the Δ(N917-P927) progesterone flooding simulation. (B) Magnification of the clustered progesterone molecules within the MtrD binding cleft.

To investigate interaction of the Δ(N917-P927) MtrD mutant with substrates, spontaneous binding simulations were performed with the substrate progesterone. To increase the likelihood of observing spontaneous substrate binding, simulations were performed with the symmetric MtrD crystal structure (PDB ID 4MT1), containing all three protomers in the A conformation, using the same protocol as previously described (23, 38). Here, 30 molecules of progesterone were randomly placed in the solution surrounding the Δ(N917-P927) MtrD porter and docking domains and triplicate 200-ns MD simulations were performed. As observed in our previous simulations (38), progesterone adsorbed nonspecifically to the protein and membrane. In two of the three simulations, multiple molecules of progesterone entered the binding cleft in the region that would have been occupied by N917-P927 (Fig. 6) and bound to the proximal binding site. In both simulations, an initial progesterone molecule first entered the binding cleft and moved into the proximal binding site before a second progesterone molecule entered the binding cleft and formed stacking interactions with the first progesterone in the proximal binding site. No closure of the binding pocket was observed in the time frame of the simulation. We postulate that deletion of N917-P927 dysregulates the access of substrate to the MtrD binding cleft and the closure of the substrate binding pocket during the transport cycle.

## DISCUSSION

In this study, we outline important features concerning multidrug export and resistance by the MtrD protein of N. gonorrhoeae involving the unique N917-P927 region. MtrD belongs to the RND superfamily of transport proteins, which in Gram-negative bacteria form one of the first line defense mechanisms against toxic chemicals, including antibiotics and host-derived antimicrobials. It has been shown that upregulation of MtrD, in synergy with other mechanisms of resistance, can result in highly drug-resistant strains of N. gonorrhoeae such as H041 and FC428 (35, 43, 44). One of the most interesting findings of this study is that the TMS 9 N917-P927 sequence is a unique dynamic region of MtrD that interacts with the membrane-periplasm interface and modulates drug access to the transporter during a functional cycle. Functional analysis of a set of MtrD mutants demonstrated that the N917-P927 region is essential for functional efflux and drug resistance capacity. In addition, MD simulations identified that this region may contribute to the positioning of the TMS 9 helix and stabilization of the protein within the neisserial IM, further facilitating efficient substrate capture.

Like AcrB, the MtrD substrate range is extremely broad and includes cationic, neutral, and anionic compounds (7, 45). Presence of a hydrophobic domain on the molecule or hydrophobicity *per se* seems to be a common feature of MtrD substrates (7, 45, 46) and suggests that they interact with the IM-periplasm interface before they are captured and exported by MtrD (45). Supporting this, RND transporters of the HAE1 family have been suggested to capture amphiphilic substrate molecules from the outer leaflet of the cytoplasmic membrane or from the membrane-periplasm interface (45, 47). Indeed, the recent MtrD crystal structures (PDB ID 6VKS and 6VKT) revealed two entrances; one from a periplasmic cleft and a second that opens to the outer leaflet of IM, both of which have been proposed as routes for hydrophobic substrate capture (24). The periplasmic end of the TMS 9 helix, including its N917-P927 region extension, is located at the membrane-periplasm boundary, such that mutations in this region are expected to impact on protein-lipid interactions in this area. As expected, MD simulations showed that deletion of the extension (ΔN917-P927) and replacement with a string of glycines [11G(917-927)] in MtrD resulted in destabilization of the protein-lipid interface and altered the positioning of MtrD within the membrane, supporting the hypothesis that this region plays a key role in stabilizing MtrD within the membrane. In addition, disturbances in the protein-lipid interface could also influence interactions with MtrD substrates at the membrane-periplasm interface and substrate capture via the IM entrance. This was supported by the reduced resistance to multiple substrates of N. gonorrhoeae cells expressing the Δ(N917-P927) MtrD mutant compared to the WT parent (Table 1). Furthermore, MD simulations of this deletion mutant with the substrate progesterone revealed that destabilization of the protein-lipid interface in the region of TMS 9 possibly impacts on substrate access to the binding cleft.

MtrD-mediated resistance to 10 selected substrates was significantly reduced by mutations to N917, G924, and P927. Their altered resistance profile could be attributed to changes in the position and relative orientation of the TMS 9 helix extension (N917-P927) during a transport cycle, since MD simulations of WT MtrD showed that the position and relative orientation of N917-P927 varies in the three protomers and involves interactions between N917, G924, and P927 with amino acids in the periplasmic domain. These variations would explain how access of hydrophobic substrates to the binding cleft entrance could be facilitated in the access protomer but efficiently prevented in the binding and extrusion protomers during a functional cycle.

In MD simulations of the N917C/G924C/P927C MtrD variant, both the position and the orientation of N917-P927 in each protomer fluctuated. In these simulations the interaction between N917-P927 and rest of the protein was most altered in the A protomer. Here, the N917-P927 region partially occluded the periplasmic substrate entrance to the binding cleft indicating that N917, G924, and P927 play a key role in substrate entry. It appears that reduced resistance capacity (Table 1) of the individual cysteine mutants (N917C, G924C, and P927C) is a result of altered interactions of these residues with rest of the protein impacting on correct positioning and orientation of N917-P927 in the membrane of N. gonorrhoeae, thereby limiting substrate access to MtrD. The first 50-ns simulations of WT MtrD revealed these important interactions. Mutations of N917, demonstrated that a positively charged polar amino acid was not tolerated in this position, since cells expressing the MtrD N917K mutant conferred resistance levels for compounds at levels only comparable to the negative control. Further analysis of the N917 asparagine side-chain demonstrated that neither glutamine nor serine (two polar noncharged residues) could compensate for asparagine, indicating that key features of asparagine are required for this position. Analysis of the residue requirement at position 927 showed that a glycine replacement in the P927G MtrD mutant still produced a fully functional protein. This supports the notion that the proline at 927 plays a structural role linked to MtrD stabilization in the IM and substrate capture from the IM-periplasm interface.

A comprehensive understanding of RND efflux pumps, which are a major contributor of antimicrobial resistance in Gram-negative bacteria, is central for combating bacterial infections, including those caused by multidrug-resistant strains of N. gonorrhoeae. Extensive genetic, biochemical, biophysical, and computational studies have hugely advanced our knowledge of efflux transporter systems and proteins, including the MtrCDE system of N. gonorrhoeae. A large body of knowledge already available with respect to the genetic aspects of the *mtrCDE* system (18), as well as the availability of crystal and cryo-EM structures of the MtrD protein in apo form (23) and in complex with drugs (24), has provided insights regarding the mechanisms used by this efflux pump to confer resistance against important antibiotics and other antimicrobials. Limited new antibiotics with activity against multidrug-resistant strains of Gram-negative pathogens have been discovered in the past 2 decades, even though they are desperately needed as this important public health problem continues to grow. Characterization of the structural and functional role of the unique N917-P927 region which connects TMS 9 and TMS 10 of MtrD provides insights concerning multidrug resistance afforded by this efflux pump. Moreover, it has revealed that subtle differences between RND transporters may have implications for drug efflux mechanisms and as such is important knowledge required to rationally design effective antibiotics or efflux pump inhibitors.

## MATERIALS AND METHODS

### Bacterial strains, plasmids, and growth conditions.

The bacterial strains and plasmids used in this study are listed in Table 2. The background N. gonorrhoeae strain is based on a FA19 derivative, KH15 that overexpresses the MtrCDE complex due to a single-base-pair deletion in the promoter of the *mtrR* gene, whose product, the MtrR protein, negatively controls expression of the *mtrCDE* operon (8, 18). KH15Δ*mtrD*Δ*norM* is a derivative of KH15 (8) with the *mtrD* and *norM* genes removed and is a highly discriminating strain for antimicrobial resistance assays (38). pGCC4-CL-*mtrD*_His6_ is a cysteine-less variant of *mtrD* (MtrD C491A) encoding a 6-histidine affinity tag at the C terminus (38). This fully functional *mtrD* derivative was used as a template for generating and mobilizing *mtrD* mutants into N. gonorrhoeae. Gonococci were grown on GC medium (Difco Laboratories, Detroit, MI) containing Kellogg’s supplements I and II (48) at 37°C under 5% CO_2_ or in GC broth (Difco Laboratories) containing Kellogg’s supplements I and II and 0.048% (vol/vol) sodium bicarbonate at 37°C with shaking. E. coli DH5α (Invitrogen, Thermo Fisher Scientific Australia Pty, Ltd.) was used for propagation of all plasmid constructs and strains were grown on Luria-Bertani (LB) agar or in LB broth (Oxoid, Thermo Fisher Scientific Australia Pty, Ltd.). Antibiotics were added at the following concentrations: kanamycin at 40 μg/ml and erythromycin at 0.5 μg/ml. Isopropyl-β-d-thiogalactopyranoside (IPTG) at a final concentration of 0.5 mM was used to induce *mtrD* transcription in N. gonorrhoeae strains harboring pGCC4 vector constructs (39). Chemicals and antibiotics, if not specified, were from Sigma Chemicals (Sigma-Aldrich Pty, Ltd.).

**TABLE 2:** Bacterial strains and plasmids used in this study

Strain or plasmid	Relevant genotypes or description	Source or reference
Strains		
E. coli		
DH5α	*fhuA2* Δ(*argF*-*lac*Z)*U169 phoA glnV*44 ϕ80 Δ(*lacZ*)M15 *gyrA96 recA1 relA1 endA1 thi-1 hsdR17*	65
N. gonorrhoeae		
KH15Δ*mtrD*Δ*norM*	KH15 is a derivative of the antibiotic-sensitive strain FA19 with a 1-bp deletion in the *mtrR* promoter with consequential *mtrCDE* upregulation and increased antibiotic resistance; has *mtrD* and *norM* genes inactivated	8, 38
		
Plasmids		
pGCC4	*Neisseria* insertion complementation system (NICS) vector; *lacI*, P_lac_; Kan^r^ Ery^r^	39
pGCC4-CL*mtrD*_His6_	Cysteine-less derivative of *mtrD*_His6_ in pGCC4	38
N917C	N917C MtrD mutant based in pGCC4-CL*mtrD*_His6_	This study
L918C	L918C MtrD mutant based in pGCC4-CL*mtrD*_His6_	This study
F919C	F919C MtrD mutant based in pGCC4-CL*mtrD*_His6_	This study
E920C	E920C MtrD mutant based in pGCC4-CL*mtrD*_His6_	This study
G921C	G921C MtrD mutant based in pGCC4-CL*mtrD*_His6_	This study
L922C	L922C MtrD mutant based in pGCC4-CL*mtrD*_His6_	This study
L923C	L923C MtrD mutant based in pGCC4-CL*mtrD*_His6_	This study
G924C	G924C MtrD mutant based in pGCC4-CL*mtrD*_His6_	This study
S925C	S925C MtrD mutant based in pGCC4-CL*mtrD*_His6_	This study
V926C	V926C MtrD mutant based in pGCC4-CL*mtrD*_His6_	This study
P927C	P927C MtrD mutant based in pGCC4-CL*mtrD*_His6_	This study
Δ(N917-P927)	917-927 deletion MtrD mutant based in pGCC4-CL*mtrD*_His6_	This study
11A(917-927)	11Ala(917-927) insertion MtrD mutant based in pGCC4-CL*mtrD*_His6_	This study
11G(917-927)	11Gly(917-927) insertion MtrD mutant based in pGCC4-CL*mtrD*_His6_	This study
N917Q	N917Q MtrD mutant based in pGCC4-CL*mtrD*_His6_	This study
N917S	N917S MtrD mutant based in pGCC4-CL*mtrD*_His6_	This study
N917K	N917K MtrD mutant based in pGCC4-CL*mtrD*_His6_	This study
P927G	P927G MtrD mutant based in pGCC4-CL*mtrD*_His6_	This study

### Molecular biology methods.

Plasmid DNA was isolated from E. coli cells using a Bioline Isolate II plasmid minikit (Bioline Meridian Bioscience, USA). Chromosomal DNA was isolated from N. gonorrhoeae cells by the use of a Wizard genomic DNA purification kit (Promega Co., USA). Primers used in this study are listed in Table S1 and were synthesized by Integrated DNA Technologies or Sigma-Aldrich. Bioline Velocity DNA polymerase was used in all PCR mutagenesis reactions and Bioline Mango*Taq* DNA polymerase for screening PCRs (Bioline, Boston, MA). All restriction digestion enzymes were from New England Biolabs (New England Biolabs, Inc., Ipswich, MA). Transformations into N. gonorrhoeae or E. coli cells were carried as described previously (38, 49). All sequencing was performed by the Australian Genome Research Facility.

### Site-directed mutagenesis.

The QuikChange site-directed mutagenesis method was used to construct *mtrD* mutants in the pGCC4-CL-*mtrD*_His6_ plasmid using the primers listed in Table S1. To aid in screening, a silent restriction site was incorporated into each primer pair where possible (38). Integrity of *mtrD* constructs were verified by sequencing the whole insert and then recombined into the FA19 KH15Δ*mtrD*Δ*norM* chromosome (38). N. gonorrhoeae mutants were assessed for mutant MtrD protein production and their resistance profile.

### Western blot analyses.

Expression of MtrD mutant proteins in N. gonorrhoeae KH15Δ*mtrD*Δ*norM* membranes was determined by Western blotting as previously described, using anti-6×Histidine epitope tag (rabbit) antibody, and peroxidase-conjugated anti-rabbit IgG (goat) (Rockland antibodies and assays) as primary and secondary antibodies, respectively (38). Mutant protein expression levels were expressed as a percentage of the wild-type protein.

### MIC analyses.

Resistance profiles of the N. gonorrhoeae KH15Δ*mtrD*Δ*norM* strain expressing the MtrD mutants were determined by MIC analyses to a selected panel of 10 compounds using standard agar dilution method (38, 50) with modifications. MIC analyses were conducted using solid GC medium supplemented with 0.5 mM IPTG and increasing concentrations of antimicrobials (Table 1). The MIC was determined as the lowest concentration of the antimicrobial compound required to fully inhibit bacterial growth and was repeated at least three times.

### MD simulations.

The ampicillin-bound MtrD cryo-EM structure (PDB ID 6VKS) was used for the WT and mutant MtrD MD simulations (23). Ampicillin was removed prior to simulations. WT and mutant MtrD simulations were carried out on four protein models: WT, Δ(N917-P927), 11G(917-927), and a triple-cysteine-replacement mutant, with concurrent mutation of residues N917, G924, and P927 (N917C/G924C/P927C). All proline residues were assigned a *trans* isomerization, consistent with the conformation observed in the cryo-EM structures. Protein mutants were generated using the PyMOL Mutagenesis Wizard and SWISS-MODEL (51). All simulations were performed using GROMACS2019.4 (52, 53) and the united-atom GROMOS 54a7 force-field (54). The MtrD protein for each system was embedded in a neisserial IM model, consisting of a 4:1 ratio of 1-palmitoyl-2-palmitoleoyl-*sn*-glycero-3-phosphatidylethanolamine (PPoPE) and 1,2-dimyristoyl-*sn*-glycero-3-phosphatidylglycerol (DMPG). Systems were oriented in the *x-y* plane of an 18-nm × 18-nm × 19-nm box, solvated with the simple-point-charge water model (55), and charge neutralized with a physiological concentration of 150 mM NaCl. GROMOS parameters for PPoPE and DMPG were obtained from previously published membrane models (56), while the parameters for ampicillin (Molid: 574595) were obtained from the Automated Topology Builder and Repository (ATB) (57, 58).

All systems were energy minimized using the steepest descent algorithm. Systems were equilibrated in a series of six sequential 2-ns simulations with progressively decreasing position restraints on the protein backbone atoms (force constants of 1,000, 500, 100, 50, 10, and 0 kJ mol^−1 ^nm^−2^). The coordinates from the final equilibration frame were used as the starting coordinates for unrestrained production simulations performed in triplicate for each system. Production simulations were 500 ns for each system.

Simulations were performed using the NPT ensemble, with solute (protein/membrane) and solvent (ions/water) separately coupled to an external temperature bath of 310K. Temperature was held constant with a Bussi thermostat (τ_T_ = 0.1 ps) (59). Pressure was maintained via semi-isotropic pressure coupling to an external pressure bath at 100,000 Pa. The Berendsen barostat was used for equilibration simulations (τ_P_ = 0.5 ps and isothermal compressibility = 4.5 × 10^−10 ^Pa^−1^) (60). Production simulations employed the Parrinello-Rahman barostat (τ_P_ = 5 ps and isothermal compressibility = 4.5 × 10^−10 ^Pa^−1^) (61). Noncovalent interactions were determined via the Verlet scheme with a 1.4-nm cutoff (62). Electrostatic interactions were calculated using Particle Mesh Ewald (63), and covalent bonds were constrained using the LINCS algorithm (64).

Progesterone flooding simulations were conducted using the symmetric apo MtrD crystal structure (PDB 4MT1) (23). Flooding simulations were carried out as previously described (28), though with the protein being changed to a Δ(N917-P927) mutant of the symmetric crystal structure generated using SWISS-MODEL (51).

## References

[1] Wi T, Lahra MM, Ndowa F, Bala M, Dillon J-AR, Ramon-Pardo P, Eremin SR, Bolan G, Unemo M. 2017. Antimicrobial resistance in *Neisseria gonorrhoeae*: global surveillance and a call for international collaborative action. PLoS Med 14:e1002344. doi:10.1371/journal.pmed.1002344. 28686231 PMC5501266

[2] Lahra MM, Martin I, Demczuk W, Jennison AV, Lee K-I, Nakayama S-I, Lefebvre B, Longtin J, Ward A, Mulvey MR. 2018. Cooperative recognition of internationally disseminated ceftriaxone-resistant *Neisseria gonorrhoeae* strain. Emerg Infect Dis 24:735–740. 10.3201/eid2404.171873 PMC5875269 29553335

[3] Shafer WM, Folster JP, Nicholas RA. 2010. Molecular mechanisms of antibiotic resistance expressed by the pathogenic *Neisseria*, p 245–267. *In* Genco CA, Wetzler L (ed), Neisseria: molecular mechanisms of pathogenesis. Caister Academic Press, Norfolk, UK.

[4] Shafer WM, Yu EW, Rouquette-Loughlin C, Golparian D, Jerse AE, Unemo M. 2016. Efflux pumps in *Neisseria gonorrhoeae*: contributions to antimicrobial resistance and virulence, p 439–470. *In* Li X-Z, Elkins CA, Zgurskaya HI (ed), Efflux-mediated antimicrobial resistance in bacteria: mechanisms, regulation and clinical implications. Springer International Publishing, Cham, Switzerland. doi:10.1007/978-3-319-39658-3.

[5] Prakash S, Cooper G, Singhi S, Saier MH, Jr. 2003. The ion transporter superfamily. Biochim Biophys Acta 1618:79–92. doi:10.1016/j.bbamem.2003.10.010. 14643936

[6] Su C-C, Bolla JR, Kumar N, Radhakrishnan A, Long F, Delmar JA, Chou T-H, Rajashankar KR, Shafer WM, Yu EW. 2015. Structure and function of *Neisseria gonorrhoeae* MtrF illuminates a class of antimetabolite efflux pumps. Cell Rep 11:61–70. doi:10.1016/j.celrep.2015.03.003. 25818299 PMC4410016

[7] Hagman KE, Lucas CE, Balthazar JT, Snyder L, Nilles M, Judd RC, Shafer WM. 1997. The MtrD protein of *Neisseria gonorrhoeae* is a member of the resistance nodulation division protein family constituting part of an efflux system. Microbiology (Reading) 143 (Pt7):2117–2125. doi:10.1099/00221287-143-7-2117. 9245801

[8] Hagman KE, Pan W, Spratt BG, Balthazar JT, Judd RC, Shafer WM. 1995. Resistance of *Neisseria gonorrhoeae* to antimicrobial hydrophobic agents is modulated by the *mtrRCDE* efflux system. Microbiology (Reading) 141 (Pt3):611–622. doi:10.1099/13500872-141-3-611. 7711899

[9] Warner DM, Shafer WM, Jerse AE. 2008. Clinically relevant mutations that cause derepression of the *Neisseria gonorrhoeae* MtrC-MtrD-MtrE Efflux pump system confer different levels of antimicrobial resistance and *in vivo* fitness. Mol Microbiol 70:462–478. doi:10.1111/j.1365-2958.2008.06424.x. 18761689 PMC2602950

[10] Maness MJ, Sparling PF. 1973. Multiple antibiotic resistance due to a single mutation in *Neisseria gonorrhoeae*. J Infect Dis 128:321–330. doi:10.1093/infdis/128.3.321. 4269625

[11] Beggs GA, Zalucki YM, Brown NG, Rastegari S, Phillips RK, Palzkill T, Shafer WM, Kumaraswami M, Brennan RG. 2019. Structural, biochemical, and *in vivo* characterization of MtrR-mediated resistance to innate antimicrobials by the human pathogen *Neisseria gonorrhoeae*. J Bacteriol 201:e00401-19. doi:10.1128/JB.00401-19. 31331979 PMC6755732

[12] Hoffmann KM, Williams D, Shafer WM, Brennan RG. 2005. Characterization of the multiple transferable resistance repressor, MtrR, from *Neisseria gonorrhoeae*. J Bacteriol 187:5008–5012. doi:10.1128/JB.187.14.5008-5012.2005. 15995218 PMC1169513

[13] Shafer WM, Balthazar JT, Hagman KE, Morse SA. 1995. Missense mutations that alter the DNA-binding domain of the MtrR protein occur frequently in rectal isolates of *Neisseria gonorrhoeae* that are resistant to faecal lipids. Microbiology (Reading) 141 (Pt4):907–911. doi:10.1099/13500872-141-4-907. 7773394

[14] Zarantonelli L, Borthagaray G, Lee E-H, Shafer WM. 1999. Decreased azithromycin susceptibility of *Neisseria gonorrhoeae* due to *mtrR* mutations. Antimicrob Agents Chemother 43:2468–2472. doi:10.1128/AAC.43.10.2468. 10508026 PMC89502

[15] Xia M, Whittington WL, Shafer WM, Holmes KK. 2000. Gonorrhea among men who have sex with men: outbreak caused by a single genotype of erythromycin-resistant *Neisseria gonorrhoeae* with a single-base-pair deletion in the *mtrR p*romoter region. J Infect Dis 181:2080–2082. doi:10.1086/315510. 10837198

[16] Cousin SL, Jr, Whittington WLH, Roberts MC. 2003. Acquired resistance genes and the 1-bp deletion in the *mtrR* promoter in *Neisseria gonorrhoeae*. J Antimicrob Chemother 51:131–133. doi:10.1093/jac/dkg040. 12493797

[17] Lindberg R, Fredlund H, Nicholas R, Unemo M. 2007. *Neisseria gonorrhoeae* isolates with reduced susceptibility to cefixime and ceftriaxone: association with genetic polymorphisms in *penA*, *mtrR*, *porB1b*, and *ponA*. Antimicrob Agents Chemother 51:2117–2122. doi:10.1128/AAC.01604-06. 17420216 PMC1891421

[18] Beggs GA, Ayala JC, Kavanaugh LG, Read TD, Hooks GM, Schumacher MA, Shafer WM, Brennan RG. 2021. Structures of *Neisseria gonorrhoeae* MtrR-operator complexes reveal molecular mechanisms of DNA recognition and antibiotic resistance-conferring clinical mutations. Nucleic Acids Res 49:4155–4170. doi:10.1093/nar/gkab213. 33784401 PMC8053128

[19] Shafer WM, Qu X, Waring AJ, Lehrer RI. 1998. Modulation of *Neisseria gonorrhoeae* susceptibility to vertebrate antibacterial peptides due to a member of the resistance/nodulation/division efflux pump family. Proc Natl Acad Sci USA 95:1829–1833. doi:10.1073/pnas.95.4.1829. 9465102 PMC19198

[20] Janganan TK, Bavro VN, Zhang L, Borges-Walmsley MI, Walmsley AR. 2013. Tripartite efflux pumps: energy is required for dissociation, but not assembly or opening of the outer membrane channel of the pump. Mol Microbiol 88:590–602. doi:10.1111/mmi.12211. 23565750 PMC3664412

[21] Tseng TT, Gratwick KS, Kollman J, Park D, Nies DH, Goffeau A, Saier MH, Jr. 1999. The RND permease superfamily: an ancient, ubiquitous and diverse family that includes human disease and development proteins. J Mol Microbiol Biotechnol 1:107–125. 10941792

[22] Saier MH, Jr, Paulsen IT. 2001. Phylogeny of multidrug transporters. Semin Cell Dev Biol 12:205–213. doi:10.1006/scdb.2000.0246. 11428913

[23] Bolla JR, Su CC, Do SV, Radhakrishnan A, Kumar N, Long F, Chou TH, Delmar JA, Lei HT, Rajashankar KR, Shafer WM, Yu EW. 2014. Crystal structure of the *Neisseria gonorrhoeae* MtrD inner membrane multidrug efflux pump. PLoS One 9:e97903. doi:10.1371/journal.pone.0097903. 24901477 PMC4046932

[24] Lyu M, Moseng MA, Reimche JL, Holley CL, Dhulipala V, Su CC, Shafer WM, Yu EW. 2020. Cryo-EM structures of a gonococcal multidrug efflux pump illuminate a mechanism of drug recognition and resistance. mBio 11:e.00996-20. doi:10.1128/mBio.00996-20. PMC7251214 32457251

[25] Seeger MA, Schiefner A, Eicher T, Verrey F, Diederichs K, Pos KM. 2006. Structural asymmetry of AcrB trimer suggests a peristaltic pump mechanism. Science 313:1295–1298. doi:10.1126/science.1131542. 16946072

[26] Sennhauser G, Amstutz P, Briand C, Storchenegger O, Grutter MG. 2007. Drug export pathway of multidrug exporter AcrB revealed by DARPin inhibitors. PLoS Biol 5:e7. doi:10.1371/journal.pbio.0050007. 17194213 PMC1717020

[27] Murakami S, Nakashima R, Yamashita E, Matsumoto T, Yamaguchi A. 2006. Crystal structures of a multidrug transporter reveal a functionally rotating mechanism. Nature 443:173–179. doi:10.1038/nature05076. 16915237

[28] Fairweather SJ, Gupta V, Chitsaz M, Booth L, Brown MH, O’Mara ML. 2021. Coordination of substrate binding and protonation in the *Neisseria gonorrhoeae* MtrD efflux pump controls the functionally rotating transport mechanism. ACS Infect Dis 7:1833–1847. doi:10.1021/acsinfecdis.1c00149. 33980014

[29] Nakashima R, Sakurai K, Yamasaki S, Nishino K, Yamaguchi A. 2011. Structures of the multidrug exporter AcrB reveal a proximal multisite drug-binding pocket. Nature 480:565–569. doi:10.1038/nature10641. 22121023

[30] Yamaguchi A, Nakashima R, Sakurai K. 2015. Structural basis of RND-type multidrug exporters. Front Microbiol 6:327. doi:10.3389/fmicb.2015.00327. 25941524 PMC4403515

[31] Eicher T, Cha HJ, Seeger MA, Brandstatter L, El-Delik J, Bohnert JA, Kern WV, Verrey F, Grutter MG, Diederichs K, Pos KM. 2012. Transport of drugs by the multidrug transporter AcrB involves an access and a deep binding pocket that are separated by a switch-loop. Proc Natl Acad Sci USA 109:5687–5692. doi:10.1073/pnas.1114944109. 22451937 PMC3326505

[32] Vargiu AV, Nikaido H. 2012. Multidrug binding properties of the AcrB efflux pump characterized by molecular dynamics simulations. Proc Natl Acad Sci USA 109:20637–20642. doi:10.1073/pnas.1218348109. 23175790 PMC3528587

[33] Sennhauser G, Bukowska MA, Briand C, Grutter MG. 2009. Crystal structure of the multidrug exporter MexB from *Pseudomonas aeruginosa*. J Mol Biol 389:134–145. doi:10.1016/j.jmb.2009.04.001. 19361527

[34] Unemo M, Golparian D, Nicholas R, Ohnishi M, Gallay A, Sednaoui P. 2012. High-level cefixime-and ceftriaxone-resistant *Neisseria gonorrhoeae* in France: novel *penA* mosaic allele in a successful international clone causes treatment failure. Antimicrob Agents Chemother 56:1273–1280. doi:10.1128/AAC.05760-11. 22155830 PMC3294892

[35] Ohnishi M, Saika T, Hoshina S, Iwasaku K, Nakayama S-I, Watanabe H, Kitawaki J. 2011. Ceftriaxone-resistant *Neisseria gonorrhoeae*. Emerg Infect Dis 17:148–149. doi:10.3201/eid1701.100397. 21192886 PMC3204624

[36] Zhang Z, Schwartz S, Wagner L, Miller W. 2000. A greedy algorithm for aligning DNA sequences. J Comput Biol 7:203–214. doi:10.1089/10665270050081478. 10890397

[37] Pos KM. 2009. Drug transport mechanism of the AcrB efflux pump. Biochim Biophys Acta 1794:782–793. doi:10.1016/j.bbapap.2008.12.015. 19166984

[38] Chitsaz M, Booth L, Blyth MT, O’Mara ML, Brown MH. 2019. Multidrug resistance in *Neisseria gonorrhoeae*: identification of functionally important residues in the MtrD efflux protein. mBio 10:e02277-19. doi:10.1128/mBio.02277-19. 31744915 PMC6867893

[39] Mehr IJ, Seifert HS. 1998. Differential roles of homologous recombination pathways in *Neisseria gonorrhoeae* pilin antigenic variation, DNA transformation, and DNA repair. Mol Microbiol 30:697–710. doi:10.1046/j.1365-2958.1998.01089.x. 10094619

[40] Rouquette-Loughlin C, Dunham SA, Kuhn M, Balthazar JT, Shafer WM. 2003. The NorM efflux pump of *Neisseria gonorrhoeae* and *Neisseria meningitidis* recognizes antimicrobial cationic compounds. J Bacteriol 185:1101–1106. doi:10.1128/JB.185.3.1101-1106.2003. 12533487 PMC142806

[41] Hassan KA, Robinson KL, Smith AN, Gibson JH, Skurray RA, Brown MH. 2006. Glycine-rich transmembrane helix 10 in the staphylococcal tetracycline transporter TetA(K) lines a solvent-accessible channel. Biochemistry 45:15661–15669. doi:10.1021/bi0614380. 17176088

[42] Hollingsworth SA, Karplus PA. 2010. A fresh look at the Ramachandran plot and the occurrence of standard structures in proteins. Biomol Concepts 1:271–283. doi:10.1515/BMC.2010.022. 21436958 PMC3061398

[43] Nakayama S-i, Shimuta K, Furubayashi K-i, Kawahata T, Unemo M, Ohnishi M. 2016. New ceftriaxone-and multidrug-resistant *Neisseria gonorrhoeae* strain with a novel mosaic *penA* gene isolated in Japan. Antimicrob Agents Chemother 60:4339–4341. doi:10.1128/AAC.00504-16. 27067334 PMC4914677

[44] Golparian D, Rose L, Lynam A, Mohamed A, Bercot B, Ohnishi M, Crowley B, Unemo M. 2018. Multidrug-resistant *Neisseria gonorrhoeae* isolate, belonging to the internationally spreading Japanese FC428 clone, with ceftriaxone resistance and intermediate resistance to azithromycin, Ireland, August 2018. Euro Surveill 23. doi:10.2807/1560-7917.ES.2018.23.47.1800617. PMC6341943 30482267

[45] Nikaido H. 1996. Multidrug efflux pumps of gram-negative bacteria. J Bacteriol 178:5853–5859. doi:10.1128/jb.178.20.5853-5859.1996. 8830678 PMC178438

[46] Nikaido H, Basina M, Nguyen V, Rosenberg EY. 1998. Multidrug efflux pump AcrAB of *Salmonella* Typhimurium excretes only those β-lactam antibiotics containing lipophilic side chains. J Bacteriol 180:4686–4692. doi:10.1128/JB.180.17.4686-4692.1998. 9721312 PMC107484

[47] Yao X-Q, Kimura N, Murakami S, Takada S. 2013. Drug uptake pathways of multidrug transporter AcrB studied by molecular simulations and site-directed mutagenesis experiments. J Am Chem Soc 135:7474–7485. doi:10.1021/ja310548h. 23627437

[48] Kellogg DS, Jr, Peacock WL, Jr, Deacon WE, Brown L, Pirkle DI. 1963. *Neisseria gonorrhoeae*. I. Virulence genetically linked to clonal variation. J Bacteriol 85:1274–1279. doi:10.1128/jb.85.6.1274-1279.1963. 14047217 PMC278328

[49] Dillard JP. 2011. Genetic manipulation of *Neisseria gonorrhoeae*. Curr Protoc Microbiol Chapter 4:Unit4A 2. doi:10.1002/9780471729259.mc04a02s23. PMC4549065 22045584

[50] CLSI. 2012. Methods for dilution antimicrobial susceptibility tests for bacteria that grow aerobically: approved standard, 9th ed, vol 32. Clinical and Laboratory Standards Institute, Wayne, PA.

[51] Waterhouse A, Bertoni M, Bienert S, Studer G, Tauriello G, Gumienny R, Heer FT, de Beer TAP, Rempfer C, Bordoli L, Lepore R, Schwede T. 2018. SWISS-MODEL: homology modeling of protein structures and complexes. Nucleic Acids Res 46:W296–W303. doi:10.1093/nar/gky427. 29788355 PMC6030848

[52] Lindahl E, Abraham M, Hess B, van der Spoel D. 2019. GROMACS 2019.4 source code. https://zenodo.org/record/3460414.

[53] Abraham MJ, Murtola T, Schulz R, Páll S, Smith JC, Hess B, Lindahl E. 2015. GROMACS: high performance molecular simulations through multi-level parallelism from laptops to supercomputers. SoftwareX 1–2:19–25. doi:10.1016/j.softx.2015.06.001.

[54] Schmid N, Eichenberger AP, Choutko A, Riniker S, Winger M, Mark AE, van Gunsteren WF. 2011. Definition and testing of the GROMOS force-field versions 54A7 and 54B7. Eur Biophys J 40:843–856. doi:10.1007/s00249-011-0700-9. 21533652

[55] Berendsen HJ, Postma JP, van Gunsteren WF, Hermans J. 1981. Interaction models for water in relation to protein hydration, p 331–342. *In* Intermolecular forces. Springer, New York, NY.

[56] Piggot TJ, Holdbrook DA, Khalid S. 2011. Electroporation of the *E. coli* and *S. aureus* membranes: molecular dynamics simulations of complex bacterial membranes. J Phys Chem B 115:13381–13388. doi:10.1021/jp207013v. 21970408

[57] Malde AK, Zuo L, Breeze M, Stroet M, Poger D, Nair PC, Oostenbrink C, Mark AE. 2011. An automated force field topology builder (ATB) and repository: version 1.0. J Chem Theory Comput 7:4026–4037. doi:10.1021/ct200196m. 26598349

[58] Koziara KB, Stroet M, Malde AK, Mark AE. 2014. Testing and validation of the Automated Topology Builder (ATB) version 2.0: prediction of hydration free enthalpies. J Comput Aided Mol Des 28:221–233. doi:10.1007/s10822-014-9713-7. 24477799

[59] Bussi G, Donadio D, Parrinello M. 2007. Canonical sampling through velocity rescaling. J Chem Phys 126:014101. doi:10.1063/1.2408420. 17212484

[60] Berendsen HJ, Postma J, van Gunsteren WF, DiNola A, Haak JR. 1984. Molecular dynamics with coupling to an external bath. J Chem Phys 81:3684–3690. doi:10.1063/1.448118.

[61] Parrinello M, Rahman A. 1981. Polymorphic transitions in single crystals: a new molecular dynamics method. J Appl Phys 52:7182–7190. doi:10.1063/1.328693.

[62] De Jong DH, Baoukina S, Ingólfsson HI, Marrink SJ. 2016. Martini straight: boosting performance using a shorter cutoff and GPUs. Comput Phys Commun 199:1–7. doi:10.1016/j.cpc.2015.09.014.

[63] Darden T, York D, Pedersen L. 1993. Particle mesh Ewald: an N · log (N) method for Ewald sums in large systems. J Chem Phys 98:10089–10092. doi:10.1063/1.464397.

[64] Hess B, Bekker H, Berendsen HJ, Fraaije JG. 1997. LINCS: a linear constraint solver for molecular simulations. J Comput Chem 18:1463–1472. doi:10.1002/(SICI)1096-987X(199709)18:12<1463::AID-JCC4>3.0.CO;2-H.

[65] Hanahan D. 1983. Studies on transformation of *Escherichia coli* with plasmids. J Mol Biol 166:557–580. doi:10.1016/s0022-2836(83)80284-8. 6345791

[66] Saier MH, Tran CV, Barabote RD. 2006. TCDB: the Transporter Classification Database for membrane transport protein analyses and information. Nucleic Acids Res 34:D181–D186. doi:10.1093/nar/gkj001. 16381841 PMC1334385

[67] Saier M. 2005 2020. Transporter classification database. http://www.tcdb.org/. Accessed 22 December 2020.

