# Supplementary material for: A Unique Sequence Is Essential for Efficient Multidrug Efflux Function of the MtrD Protein of Neisseria gonorrhoeae
Source: mBio. 2021 Aug 31;12(4):e01675-21. doi: 10.1128/mBio.01675-21 (PMC8406276; doi:10.1128/mBio.01675-21)
Supplement: TABLE S1 [file mbio.01675-21-st001.docx]

## Supplemental data

**TABLE S1** Oligonucleotides used in this study

| **Name and purpose** | **Sequence 5' to 3'** | **Source** |
| --- | --- | --- |
| PCR screening for integration of *mtrD* between *aspC* and *lctP* | | |
| SCRNG1 | CGCCTTATGATGCAAATG | ([38](#_ENREF_38)) |
| MTRDSF5 | CCGTATTCTGGGGGATGTTG | ([38](#_ENREF_38)) |
| Sequencing of *mtrD* in pGCC4 | | |
| PGCC4_F2 | CCCAGGCTTTACACTTTATGCT | ([38](#_ENREF_38)) |
| PGCC4-R1.1 | GGGGATCCGCTAGCACTAGG | ([38](#_ENREF_38)) |
| MtrD-SF4.1 | GCAGTTGGGTACGGCAGAAG | ([38](#_ENREF_38)) |
| MtrD-SR4.1 | GCTCTGTTCCGTACCGTTTTC | ([38](#_ENREF_38)) |
| Mutagenesis | | |
| MTRD-L F1 | GGGCGTAACCGGTCGCTCGTTTGCCAACGACATC | This study |
| MTRD-L R1 | GATGTCGTTGGCAAACGAGCGACCGGTTACGCCC | This study |
| DLT92_11Ala_F | CTGCGGGCGTAACCGGGCGCGCTGCAGCTGCAGCCGCGGCTGCAGCTGCAGCCTCGTTTGCCAACGACATC | This study |
| DLT92_11Ala_R | GATGTCGTTGGCAAACGAGGCTGCAGCTGCAGCCGCGGCTGCAGCTGCAGCGCGCCCGGTTACGCCCGCAG | This study |
| DLT92_11Gly_F | CTGCGGGCGTAACCGGGCGCGGTGGAGGTGGAGGTGGGGGTGGAGGTGGAGGCTCGTTTGCCAACGACATC | This study |
| DLT92_11Gly_R | GATGTCGTTGGCAAACGAGCCTCCACCTCCACCCCCACCTCCACCTCCACCGCGCCCGGTTACGCCCGCAG | This study |
| N917C For | GCGTAACCGGGCGCTGCCTGTTTGAAGGACTGTTGG | This study |
| N917C Rev | CCAACAGTCCTTCAAACAGGCAGCGCCCGGTTACGC | This study |
| L918C For | CGTAACCGGGCGCAACTGCTTCGAAGGACTGTTGG | This study |
| L918C Rev | CCAACAGTCCTTCGAAGCAGTTGCGCCCGGTTACG | This study |
| F919C For | CGGGCGCAACCTGTGTGAAGGACTGTTGG | This study |
| F919C Rev | CCAACAGTCCTTCACACAGGTTGCGCCCG | This study |
| E920C For | GGCGCAACCTGTTTTGCGGTCTCTTGGGCAGCGTTC | This study |
| E920C Rev | GAACGCTGCCCAAGAGACCGCAAAACAGGTTGCGCC | This study |
| G921C For | GCAACCTGTTTGAATGTCTTCTGGGCAGCGTTCC | This study |
| G921C Rev | GGAACGCTGCCCAGAAGACATTCAAACAGGTTGC | This study |
| L922C For | GCAACCTGTTTGAAGGATGCTTAGGCAGCGTTCCCTC | This study |
| L922C Rev | GAGGGAACGCTGCCTAAGCATCCTTCAAACAGGTTGC | This study |
| L923C Fw | CAACCTGTTTGAAGGCCTGTGCGGCAGCGTTCCCTCG | This study |
| L923C Rev | CGAGGGAACGCTGCCGCACAGGCCTTCAAACAGGTTG | This study |
| G924C Fw | CAACCTGTTTGAAGGACTGCTCTGCAGCGTTCCCTCGTTTGC | This study |
| G924C Rev | GCAAACGAGGGAACGCTGCAGAGCAGTCCTTCAAACAGGTTG | This study |
| S925C Fw | CCTGTTTGAAGGCCTGTTGGGCTGCGTTCCCTCGT | This study |
| S925C Rev | ACGAGGGAACGCAGCCCAACAGGCCTTCAAACAGG | This study |
| V926C Fw | AGGACTGTTGGGCAGCTGTCCCTCGTTTGCCAAC | This study |
| V926C Rev | GTTGGCAAACGAGGGACAGCTGCCCAACAGTCCT | This study |
| P927C Fw | ACCTGTTTGAAGGACTGTTGGGATCCGTTTGCTCGTTTGCCAACGACATCTAC | This study |
| P927C Rev | GTAGATGTCGTTGGCAAACGAGCAAACGGATCCCAACAGTCCTTCAAACAGGT | This study |
| N917K F | CTGCGGGCGTAACCGGTCGCAAACTGTTTGAAGGACTGTTGG | This study |
| N917K R | CCAACAGTCCTTCAAACAGTTTGCGACCGGTTACGCCCGCAG | This study |
| P927G F | CAACCTGTTTGAAGGACTGTTGGGATCCGTTGGCTCGTTTGCCAACGACATCTAC | This study |
| P927G R | GTAGATGTCGTTGGCAAACGAGCCAACGGATCCCAACAGTCCTTCAAACAGGTTG | This study |
| N917Q F | GTGCGGCTGCGGGCGTAACCGGTCGACAGCTGTTTGAAGGACTGTTGGGCAGCGTTC | This study |
| N917Q R | GAACGCTGCCCAACAGTCCTTCAAACAGCTGTCGACCGGTTACGCCCGCAGCCGCAC | This study |
| N917S F | GTGCGGCTGCGGGCGTAACCGGGCGATCGCTGTTTGAAGGACTGTTGGGCAGCGTTC | This study |
| N917S R | GAACGCTGCCCAACAGTCCTTCAAACAGCGATCGCCCGGTTACGCCCGCAGCCGCAC | This study |
