# Supplementary figures and images for: A Unique Sequence Is Essential for Efficient Multidrug Efflux Function of the MtrD Protein of Neisseria gonorrhoeae
Source: mBio. 2021 Aug 31;12(4):e01675-21. doi: 10.1128/mBio.01675-21 (PMC8406276; doi:10.1128/mBio.01675-21)

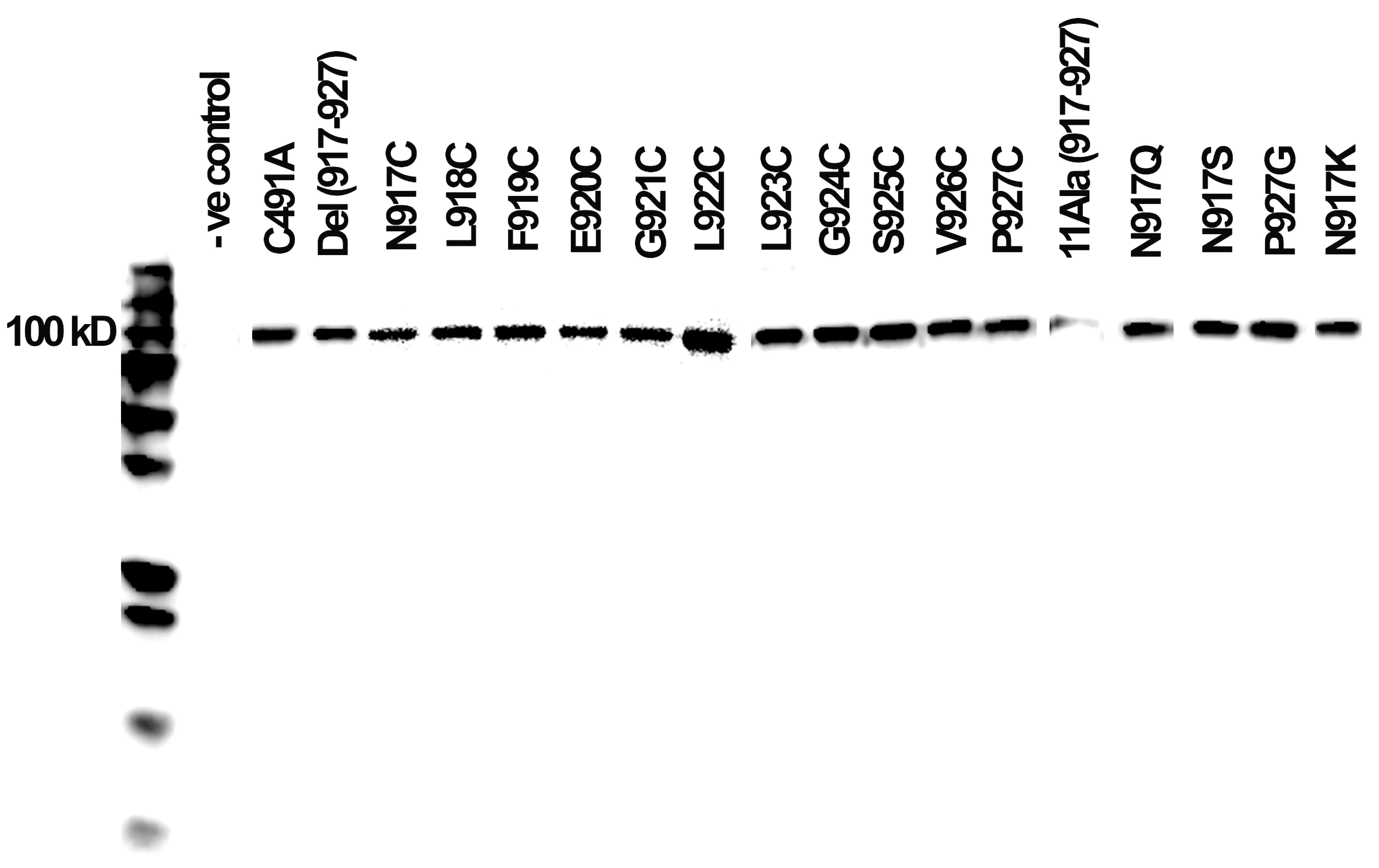

Supplement: FIG S1 [file mbio.01675-21-sf001.tif]
